# Er:YAG laser-induced cavitation can activate irrigation for the removal of intraradicular biofilm

**DOI:** 10.1038/s41598-022-08963-x

**Published:** 2022-03-22

**Authors:** Taiji Nagahashi, Yoshio Yahata, Keisuke Handa, Masato Nakano, Shigeto Suzuki, Yusuke Kakiuchi, Toshinori Tanaka, Masafumi Kanehira, Venkata Suresh Venkataiah, Masahiro Saito

**Affiliations:** 1 Division of Operative Dentistry, Department of Ecological Dentistry, Tohoku University Graduate School of Dentistry, 4-1 Seiryo-machi, Aoba-ku, Sendai, Miyagi 980-8575 Japan; 2 Division of Molecular Biology and Oral Biochemistry, Department of Oral Science, Graduate School of Dentistry, Kanagawa Dental University, 82 Inaoka-cho, Yokosuka, Kanagawa 238-8580 Japan

**Keywords:** Endodontics, Root canal treatment, Preclinical research

## Abstract

We investigated the biofilm removal effects of laser activated irrigation (LAI) using a pig model, focusing on the impact of the fiber tip position, and used a high-speed camera to observe the occurrence and positioning of the cavitation associated with laser irradiation. A total of 16 roots of deciduous mandibular second premolars from 4 pigs were used. After a pulpectomy, the canals were left open for 2 weeks and sealed for 4 weeks to induce intraradicular biofilm. Root canal irrigation was then performed with Er:YAG laser activation. The fiber tip was inserted at two different positions, i.e., into the root canal in the intracanal LAI group and into the pulp chamber in the coronal LAI group. Intracanal needle irrigation with saline or 5% NaOCl was utilized in the positive control and conventional needle irrigation (CNI) groups. SEM and qPCR were carried out to evaluate treatment efficacy. Statistical analysis was performed using ANOVA and a Tukey–Kramer post-hoc test for qPCR and with a Steel–Dwass test to compare the SEM scores, with α = 0.05. A high-speed camera was used to observe the generation of cavitation bubbles and the movement of the induced bubbles after laser irradiation. The intracanal and coronal LAI groups showed significantly lower amounts of bacteria than either the positive control or CNI groups. There was no significant difference found between the intracanal and coronal LAI groups. SEM images revealed opened dentinal tubules with the destruction of biofilm in both LAI groups. High-speed camera images demonstrated cavitation bubble production inside the root canal after a single pulse irradiation pulse. The generated bubbles moved throughout the entire internal multi-rooted tooth space. Coronal LAI can generate cavitation in the root canal with a simply placed fiber inside the pulp chamber, leading to effective biofilm removal. This method could thus contribute to the future development of endodontic treatments for refractory apical periodontitis caused by intraradicular biofilm.

## Introduction

Apical periodontitis refers to purulent inflammation inside the alveolar bone triggered by a bacterial infection of the root canal system, which is the internal structure of the tooth^1^. Treatments require the removal of causative bacteria and their by-products from the root canal system and suppression of the inflammatory processes. Mechanical and/or chemical bacterial removal have been utilized as standard approaches in these cases under an aseptic environment, and the success rates for these initial root canal treatments have been reported to exceed 90%^2^. Retreatments are required in cases of refractory inflammation, but the success rates will typically be lower^3^ because of bacterial ingrowth into anatomical complexities such as the lateral canal and isthmus and the subsequent formation of biofilm^4^. The complete elimination of biofilm is almost impossible in this circumstance.

Root canal irrigation is a treatment technique that aims to chemically reduce bacterial loads inside the root canal system, such as in areas that cannot be reached by a mechanical root canal preparation. The standard irrigation method utilizes a syringe and fine needle (i.e., 27–31 G) to deliver and reflux the disinfectant solution. Recently, irrigant agitation techniques using ultrasound, sonic vibrations, and lasers have been shown to be more effective in reducing intracanal debris than the syringe technique^5–8^. Notably however, removing bacterial biofilm from the root canal surface has remained an issue with these interventions because chemical actions alone are insufficient to remove biofilm and some form of physical manipulation is also needed. Hence, although the chemical exposure from a root canal irrigation will be effective against planktonic bacteria, irrigation technologies are needed that can eliminate biofilm through the generation of physical force at an adjacent site that is inaccessible to existing mechanical instruments. Among the various types of root canal irrigation techniques that are currently available, photodynamic actions are a possible approach to generating a physical effect during root canal irrigation by means of hydrodynamic force generation.

Photodynamic actions produced by multiple lasers at different wavelengths have been shown to effectively agitate root canal irrigants. An Er:YAG laser emitting at the 2.94 µm wavelength close to the absorption peak of water^9,10^, has been utilized for Laser-activated irrigation (LAI), which effectively removes bacteria from the root canal system^11^. The LAI cleaning mechanism depends on rapid fluid motion^10,12,13^ in the root canal, which generates subsequent pressure waves through the expansion and collapse of vapor bubbles at the site of the laser irradiation. In addition, the generation of numerous secondary cavitation bubbles can be observed under a high-speed camera after the vapor bubble collapse^14^. Cavitation is a liquid to gas phase transformation phenomenon caused by a decreased pressure due to an increased fluid velocity. A collapse of the cavitation bubbles occurs after this and produces large-amplitude shock waves. Hence, LAI is expected to have properties that not only increase the flow velocity of the irrigant but also generate physical forces upon cavitation collapse on the root canal wall that could be effective for biofilm removal. Since laser agitation of the root canal irrigant can spread throughout the root canal away from the fiber tip, placement of the fiber tip into the pulp chamber without coming close to the apex or into the root canal has been advocated, referred to as photon-induced photoacoustic streaming (PIPS)^15,16^. In addition, new laser cleaning technologies are being developed, such as SWEEPS, which enables the generation of shockwaves^17^. PIPS and SWEEPS are characterized by the use of a shorter pulse duration such as 50 µsec, compared to that of conventional LAI (300 µs). On the other hand, a previous study has reported that biofilm removal was possible with conventional pulse-width LAI^18^. The mechanisms of biofilm removal by LAI with a longer pulse duration are still unclear however.

While the usefulness of root canal irrigation using an Er:YAG laser has been pointed out in many prior reports, most of these previous studies have been conducted in vitro or ex vivo, and few in vivo or clinical studies have been conducted to date^16^. Also, in studies that have compared biofilm removal properties, the composition of the biofilm becomes an issue. In many investigations, the biofilm was composed of a monospecies only, which is different from the actual clinical situation^19^. The multispecies biofilm removal capacity of a laser is unknown. In our present study therefore, we used a pig intraradicular biofilm model to investigate the effects of laser irradiation on biofilm removal, with a particular focus on the impact of the fiber tip position. We also employed a high-speed camera to carefully observe the occurrence and positioning of the cavitation associated with the laser irradiation.

## Methods

The current study protocols for animal use were reviewed and approved by the Animal Care and Use Committees of Tohoku University (Permit No. 2018 SHIDO-045). All animal experiments were conducted in accordance with the Regulations for Animal Experiments and Related Activities at Tohoku University.

**Figure 1:**
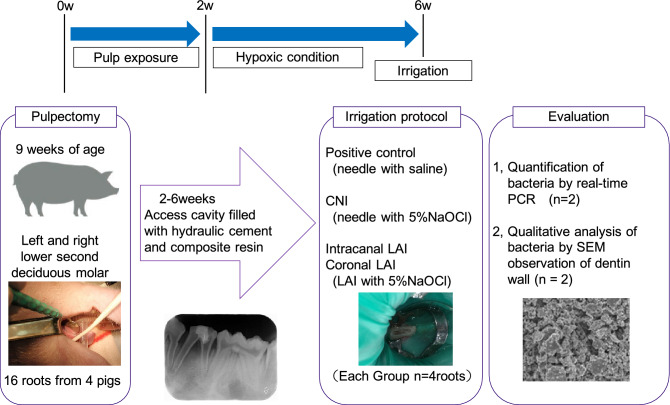
Experimental outline for the current study in the pig model. After the removal of the pulp tissue, the canal was exposed to the oral environment for 2 weeks and to an anaerobic intracanal environment for 4 weeks to allow for bacterial biofilm maturation. Six weeks after pulp removal, the pig teeth were irrigated by one of four different test protocols. Qualitative evaluations by SEM and quantitative assessment by qPCR were then performed. CNI, conventional needle irrigation; LAI, laser activated irrigation.

Sixteen roots from the deciduous mandibular second premolars of four pigs (9-week-old large white X Landrace breed cross; Japan SLC Inc., Shizuoka, Japan) were included. The light was turned on at 8.00 a.m. and turned off at 6.00 p.m. each day in our animal facility. The pigs had free access to water at any time and were fed a regular diet (Grandeal B; Zennoh Feed Mills of the Tohoku District, Miyagi, Japan) 3 times per day. All interventions were performed after sedation with medetomidine (0.1 mg/kg, IM) and midazolam (0.2 mg/kg, IM) followed by inhaled sevoflurane (2–5%), with local injections of 2% lidocaine (1.8 mL, SC) also given to minimize pain. The experimental protocol is presented in Fig. 1. All procedures were performed under surgical loupes with LED light (EyeMag PRO; Carl Zeiss, Jena, Germany).

An intracanal biofilm was induced in the animals following a previously reported procedure^18^. Briefly, the occlusal surface was flattened using a straight bur and electric engine (Ti-Max X95; NSK, Tochigi, Japan) to prevent tooth fracture and for the ease of working length determination. Following access cavity preparation and after obtaining a straight-line access, chemo-mechanical removal of pulp tissue was performed with 5% sodium hypochlorite (NaOCl) and K files. The canals were exposed to the oral environment to inoculate the root canal with oral bacteria for 2 weeks. This was followed by sealing with hydraulic temporary filling material (Lumicon; Heraeus Kulzer, South Bend, IN, USA) and composite resin (MI Flow II; GC, Tokyo, Japan) with the use of adhesive (G-Premio BOND, GC) to create an anaerobic intracanal environment for 4 weeks to allow the bacterial biofilm to mature. After removal of the temporary filling materials and placement of the dental dam isolation at 6 weeks after pulp removal, aseptic conditions were established by cleaning the tooth surface with 5% NaOCl and saline. The teeth were then randomly assigned to the different experimental irrigation groups.

The following irrigation protocols were used for the different experimental groups.

CNI group: conventional needle irrigation (CNI) was performed using side port 30G needles with 5% NaOCl. Canals were irrigated for 30 s with 5 mL of irrigant and left for 30 s. This cycle was repeated five times for a total of five minutes of irrigation.

Intracanal LAI (I-LAI) group: irrigants were activated using an Er:YAG laser (Erwin AdvErl EVO; Morita, Kyoto, Japan) using a cone-shaped 300 µm diameter tip (R300T). Canals were irrigated for 30 s with 5 mL of the solution using a side port 30G needle and the irrigants were activated for LAI with the laser settings at 30 mJ and 20 pps without air and water supply. This cycle was repeated five times for a total of five minutes of irrigation and subsequent agitation. During laser irradiation, the tip was inserted 2 mm short of the working length and was moved slowly up and down a 3 mm length to the coronal side as with the conventional LAI irrigation.

Coronal LAI (C-LAI) group: this irrigation protocol had the same conditions as the I-LAI group except for the laser fiber tip position, which was inside the pulp chamber and kept stationary. During laser irradiation, the solution in the pulp chamber maintained the volume, so that a light-cured resin built up around the crown (Dentto-Dam, MEDICLUS, Korea), and NaOCl was then added with a syringe.

The laser output parameters of I-LAI and C-LAI were identical. Briefly, the transmitting efficiency of the optical fiber and R300T tip was 90% and 40%, respectively. The average output power was 0.22 W when using the 30 mJ, 20 pps output conditions in this experiment. The peak power was 36.7 W since the pulse duration was 300 μsec. The irradiation area of the R300T tip was 300 μm, and the total irradiation time was 150 s. Hence, the power density and total energy were 73 W/cm^2^ and 33 J, respectively, for activation of the irrigation.

Positive control group: the teeth irrigated using saline were used as the positive control group.

After root canal irrigation, each canal was rinsed with saline for 30 s and sealed with hydraulic temporary filling material (Lumicon). After cleaning with a 10% povidone-iodine antiseptic solution, the teeth were extracted, and the crown was resected. Thus, two roots were taken from one tooth, one of which was sampled for real-time PCR analysis (n = 2 in each group) and one for SEM (n = 2 each group). The roots were assigned to these experiment randomly. All surgical instruments and bars were sterilized before use.

Quantifications of the bacteria present in the root canals were performed based on previously described methods. Briefly, the sample roots were immersed in liquid nitrogen immediately after the tooth crown was resected. For bacterial quantification, an intact deciduous mandibular second premolar, to be used as the sound tooth, was obtained in the same manner as the other specimens from a pig used for other purposes. Two roots from the sound tooth were preserved as an intact root canal with no exposure to bacteria or any root canal procedure. The roots were then crushed using a sterilized SK mill (Tokken, Chiba, Japan) to acquire powdered samples. Total DNA was extracted from each powdered root sample using a Cica Geneus DNA extraction Kit (Kanto Chemical Co., Tokyo, Japan) in accordance with the manufacturer’s instructions. The presence of bacteria was verified in the experimental samples by qPCR using the bacterial primers 357F and 908R22. These assays were performed using a real-time PCR apparatus (CFX Connect; Bio-Rad Laboratories, Hercules, CA, USA). Amplifications were conducted for 40 cycles at 95 °C for 15 s followed by 65 °C for 1 min, with the fluorescence signals measured at the end of each cycle. A standard curve was generated by subjecting tenfold dilutions of a known concentration of *E. faecalis* DNA to the same qPCR protocol. The bacterial counts in all experimental groups were calculated using threshold cycle (Ct) values plotted against the standard curve.

SEM sample preparation was conducted according to previously described methods^18,20^. Briefly, after resecting the tooth crown, the mesial and distal roots were separated with a diamond disc. The distal root was then split into halves and immersed in 2.5% glutaraldehyde to fix the root canal biofilm, and then rinsed with PBS and treated with 1-ethyl-3-methyl-imidazolumetrafluoroborate. The samples were then dried in a vacuum desiccator for one day and sputter-coated with platinum. The surfaces of the root canal wall and intraradicular biofilm were observed using SEM (VE-8800; Keyence Inc., Osaka, Japan) at a 10 kV acceleration. In each sample, 2 images were taken randomly within the middle third of the root. The SEM images were scored for the presence of debris and a smear layer according to a previously described scale^21^. Two evaluators conducted this scoring independently and any differences were resolved by discussion. The evaluators and the technician generating the SEM images were blind to any information regarding the specimens and had no involvement in the rest of the experiment. The presence of debris was evaluated and scored under 30–1000× magnification as follows: (1) clean root canal wall; (2) a few small agglomerations of debris; (3) many debris agglomerations covering less than 50% of the canal wall; (4) more than 50% of the canal wall covered by debris; (5) nearly complete root canal wall covered by debris. The presence of a smear layer was evaluated and scored under 1000–3000× magnification as follows: (1) All dentinal tubules open; (2) 25–75% of dentinal tubules open; (3) < 25% of dentinal tubules open, a homogeneous smear layer covering the canal wall; (4) no dentinal tubules open, a homogeneous smear layer completely covering the canal wall; (5) a heavy, inhomogeneous smear layer covering the canal wall.

To observe the generation and movement of bubbles inside the root canal through the LAI, images were taken using a high-speed digital camera (FASTCAM Mini AX200; Photron, Tokyo, Japan) attached to a macro lens (SP 90 mm F/2.8 Di; Tamron, Saitama, Japan). The LED light source was placed diagonally in front of the root canal model. The Er: YAG laser was equipped with R300T fiber tip with an output setting of 30 mJ at 20 PPS and no water or air supply.

Two types of root canal models were used i.e., a single or two canal model. A single root canal model made of epoxy resin with an apical size #80, 02 taper, and length of 15 mm was used to observe the characteristics of the bubbles generated with a laser pulse. The fiber tip was placed 12 mm from the apex, and the root canal was filled with water. The frame rate, image size, and recording duration were set at 40,000 frames per sec (fps), 384 × 256 pixels, and 217 ms, respectively. For the two root canal model, an artificial maxillary premolar model (TrueTooth # 5-002, Dental Engineering Laboratories, CA, USA) was used to observe the induction of bubbles and changes in their behavior over time. The tooth was prepared with an access opening, straight-line access, and root canal preparation with a size #50 Reciproc Blue (VDW, Munich, Germany). The fiber tip was placed at the center of the pulp chamber as assuming C-LAI. During laser irradiation, the irrigant was continuously supplied with a syringe. The frame rate, image sizes, and recording duration were set at 750 fps, 1024 × 1024 pixels, and 29 s, respectively.

Statistical analysis was performed using ANOVA followed by a Tukey–Kramer post-hoc test to detect differences in bacterial counts and with a Steel–Dwass test to compare the SEM scores. Data were compared using BellCurve for Excel statistical software (Social Survey Research Information Co., Ltd. Tokyo, Japan) with an α value of 0.05.

### Ethical approval and consent to participate

This study was reviewed and approved by the Animal Care and Use Committees of Tohoku University Graduate school of Dentistry (Permit No. 2018 SHIDO-045). Animal studies followed the ARRIVE 2.0 guidelines. All applicable international, national, and/or institutional guidelines for the care and use of animals were adhered to. Consent to participate was not applicable.

## Results

**Figure 2:**
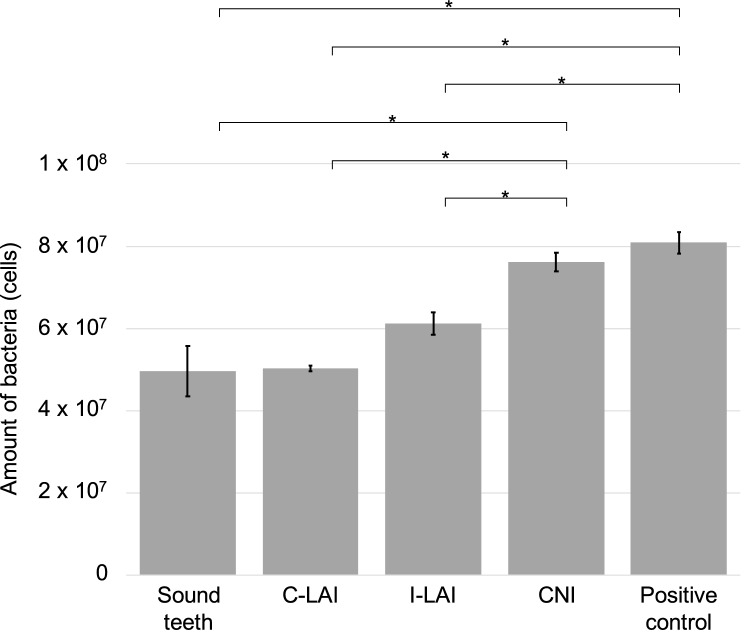
Quantitative evaluation of the number of bacteria in the root canal by qPCR. The I-LAI and C-LAI groups had a significantly lower bacterial concentration than either the positive control or CNI groups. There was no significant difference found between the I-LAI and C-LAI groups. The number of bacteria in the positive control and CNI groups was significantly higher than that in the sound teeth. However, the bacterial levels in both LAI groups were not significantly different from those in the sound teeth.

Figure 2 shows the qPCR results representing the remaining bacterial amounts in the root canal after experimental root canal irrigation. The I-LAI group (6.11 × 10^7^) and the C-LAI group (5.02 × 10^7^) showed a significantly lower level of bacteria than either the positive control group (8.08 × 10^8^) or the CNI group (7.62 × 10^7^). There was no significant difference found between the I- and C-LAI groups in this regard. Compared to the sound teeth (4.95 × 10^7^), the bacterial counts in the positive control group and the CNI group were significantly higher, while those in the both LAI groups did not differ significantly.

**Figure 3:**
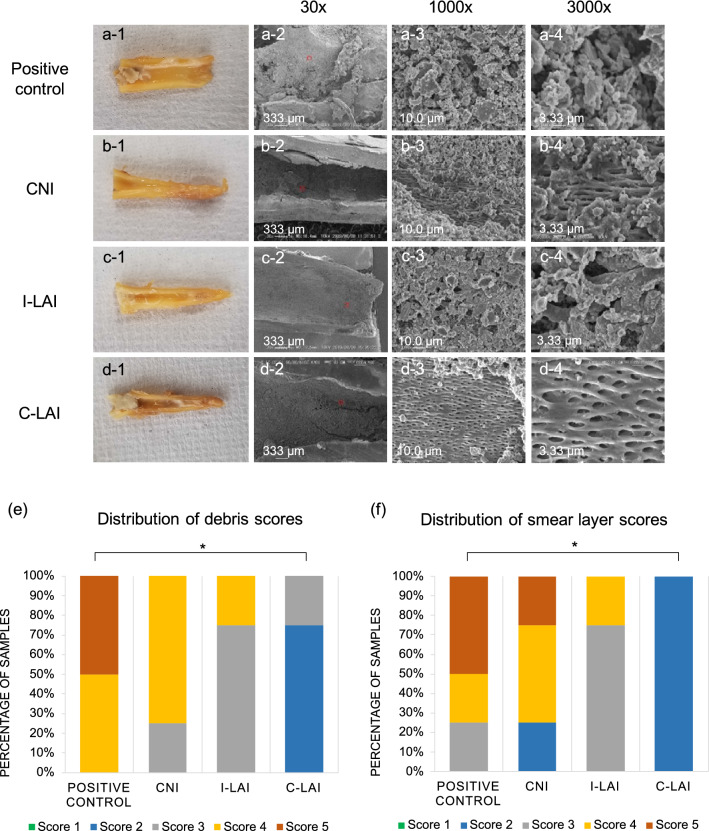
Typical images (**a-1**, **b-1**, **c-1**, and **d-1**) and SEM images (×30: **a**, **b**, **c**, and **d-2**; ×1000: **a**, **b**, **c**, and **d-3**; ×3000: **a**, **b**, **c**, and **d-4**) from each study group. In the high magnification image, a residual layer of biofilm was observed to cover the root canal wall in the positive control group, and the structure of the root canal wall could not be confirmed (**a-4**). In the CNI and I-LAI groups, some openings of the dentinal tubules were observed compared with the positive control group, but some biofilm remained (**b-4**, **c-4**). In the C-LAI group however, almost no biofilm remained, and opening of both the dentinal tubules and the reticulated intertubular dentin structure could be observed (**d-4**). The distributions of the scores for debris (**e**) and smear layer (**f**) are shown. In both instances, the C-LAI group showed significantly better scores than the positive control.

SEM images of the root canal wall taken after root canal irrigation are shown in Fig. 3a–d. A multi-layered biofilm that covered the entire root canal was observed in the positive control group. Some dentinal tubule structures could be seen in the CNI and I-LAI groups due to biofilm destruction, although residual biofilm was observed. Only a small amount of biofilm remained in the C-LAI group, and dentin tubule structures could be seen on the entire root canal wall of these teeth. Figure 3e,f show the distributions of both the debris and smear layer scores. None of the specimens were categorized as having a complete removal of debris or smear layer. In both sets of scores, C-LAI scored significantly better than the positive control, whereas no other group comparisons showed significant differences.

**Figure 4:**
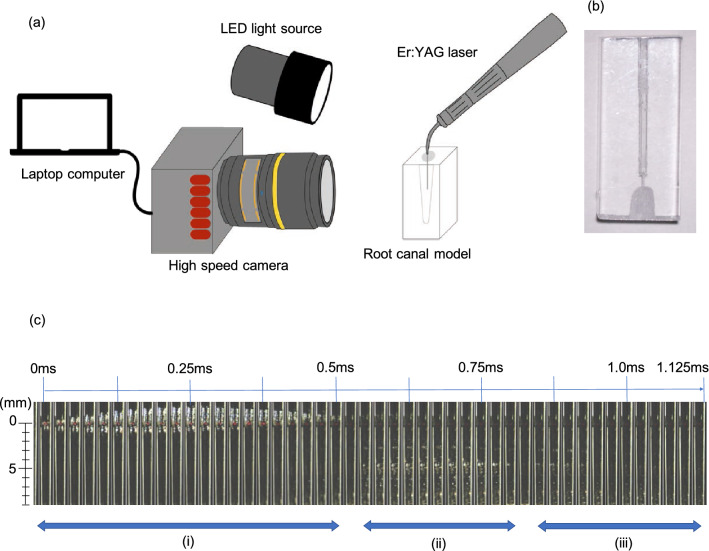
(**a**) Settings used for the observation of bubble behavior in a single pulse with a high-speed camera. (**b**) Single canal model. (**c**) Captured images showing the generation and disappearance of vapor bubbles at the tip, and the generation of cavitation bubbles. Recording conditions were set at 40,000 fps, 384 × 256 pixels, and 217 ms. At the tip, the bubbles generated at t = 0 ms notably developed in the crown direction, shrank, and finally disappeared at t = 0.525 ms (i). Additionally, several cavitation bubbles could be observed from t = 0.625 ms to t = 0.825 ms at the central part of the canal (ii). The cavitation bubble was observed to generate and disappear again within the same time range from 0.875 ms to 1.15 ms (iii).

Bubble generation and collapse after a single pulse of irradiation inside a single root canal model is demonstrated in Fig. 4. A supplementary movie file shows in more detail (see Supplementary Video 1). At the tip, the bubbles generated at t = 0 ms most notably developed in the crown direction, shrank, and then finally disappeared at t = 0.525 ms. Additionally, several cavitation bubbles were observed from t = 0.625 ms to t = 0.825 ms at the central part of the canal appearing at around 3 to 8 mm from the fiber tip. Subsequently, cavitation bubbles were repeatedly observed in the same range indicated above from 0.875 ms to 1.15 ms. The bubble at the fiber tip was found to have shrunk the most at t = 0.5 ms, with a bubble appearing at a distance of about 5 mm approximately 0.05 ms later. We surmised from this that bubbles are generated in the tubules by the pressure wave generated during their shrinking and re-expansion at the tip end, in addition to the associated depressurization. In this case, the velocity of the pressure wave can be calculated to be about 100 m/sec.

**Figure 5:**
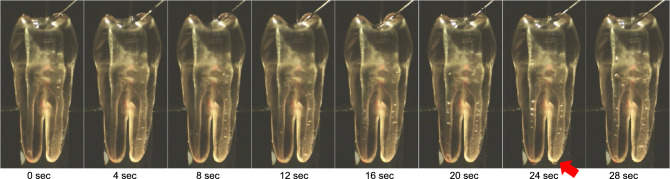
Observation of bubble behavior over time in the two root canal model. The number and size of the bubbles in the root canal increased with time from 0 s at the time of laser irradiation, and bubbles were observed over a wide range from the coronal area to the apex of both root canals. A slight overflow of the solution was observed from the apex at 24 s (red arrow). Recording conditions were set at 750 fps, 1024 × 1024 pixels, and 29 s.

The movement behavior of the bubbles over 30 s is depicted in Fig. 5 and in the supplementary movie file (see Supplementary Video 2). After laser irradiation, the bubbles generated in the pulp chamber reached the root canal orifice in 1.03 s and the root apex in 1.75 s in the left root canal. In the right root canal, the bubbles reached the root canal orifice in 1.15 s and the root apex in 7.64 s. The number and size of the bubbles in the root canal increased with time. The generated bubbles and their movement could be observed across the entire internal tooth space. A slight irrigant extrusion from the root apex was observed at t = 24 s.

## Discussion

Removing biofilm from inside a root canal, which is the cause of refractory apical periodontitis, is still challenging when conducting a root canal retreatment^4,22^. The most reliable biofilm removal method is mechanical disruption, although no instrumentation technique to achieve this can reach all of the root canal surfaces^23^. In addition, biofilms are observed in anatomical complexities such as the isthmus and lateral canal^24^. Hence, non-contact biofilm removal techniques continue to need a more reliable retreatment protocol. We have previously reported an in vivo intraradicular biofilm model in the pig^18^, which can closely replicate human biofilms in terms of morphology and microbiota, to evaluate the effectiveness of biofilm removal protocols and found that LAI is an effective intervention in this regard. The Er:YAG laser is immediately absorbed by water upon irradiation and generates bubbles that create pressure waves which will agitate the cleaning solution via the high-velocity irrigant flow^8,13,14,25^. LAI was found previously to be significantly more effective in cleaning root canals, especially in removing root canal debris and intracanal bacteria, than CNI or ultrasonic agitation^11^. Moreover, the vapor bubble- and cavitation bubble-induced turbulence associated with LAI can cause shearing stress on the root canal wall surface^11,26^. In our present study, C-LAI showed the capacity to remove biofilm along with excellent cleaning effects, which indicated that LAI has the potential to apply the non-contact removal technique.

The results of our bacterial quantification analysis revealed that laser irradiation is effective in reducing the bacterial content, yet a certain amount of bacteria was also found in the sound tooth, which should be intact and almost aseptic as the root canal. However, the frozen tooth root samples analyzed by qPCR included the root surface, and bacteria on this surface may have been detected. As reported previously^18^, the oral hygiene of pigs is generally poor, and even if the surfaces are disinfected before tooth extraction, it is difficult to completely remove all bacteria. In addition, contamination may occur during procedures such as tooth extraction and root crushing, although tooth disinfection and sterilized instruments were used in our protocols. In order to elucidate the impact of these contaminations, a sound tooth was employed as a negative control for quantification of bacterial counts. Hence, the same amount of bacteria were counted following C-LAI and I-LAI as in the sound tooth, indicating that the cleaning was effective.

The fiber tip used in our current investigations has a conical tip and is designed to distribute 80% of the irradiated laser energy laterally and 20% axially. When such a conical-shaped tip is inserted into the root canal system, regarded as a I-LAI approach, the space between the fiber tip and the root canal wall is insufficient to induce irrigant flow. Most of the irradiated energy in this case goes directly to the root canal wall^14,27^. On the other hand, the C-LAI or PIPS methods reserve a sufficient amount of space because the fiber tip is placed into the pulp chamber so that the energy is absorbed by the irrigant and can therefore produce a high irrigant velocity^28^. In our present study, we found that a C-LAI can simultaneously observe the generation of bubbles and disperse them throughout the whole root canal system. Also, the extent of the bacterial reduction by C-LAI is comparable to that of I-LAI, indicating that C-LAI can achieve a sufficient flow velocity to agitate the solution in the entire root canal system. The effect of I-LAI was limited to the periphery of the tip, resulting in the presence of biofilm remnants in SEM observations. In our present experiments, we inserted the fiber tip at 2 mm shorter than the working length of the I-LAI, although many prior studies have done so at 5 mm shorter^13,27,29,30^. Another study observed the effective irrigant flow around the apex when the conical shaped tip was positioned at 2 mm from the apex^14^, similar to our present study. In our present study therefore, the tip was inserted as close as 2 mm to the apex in order to achieve a sufficient cleaning effect. On the other hand, inserting the tip close to the apex increases the risk of the irrigant leaking out of the apex. As for C-LAI, the leakage of solution beyond the root apex was observed in the two root canal model. Additional research is needed to ensure safety, yet the time required between the irradiation and the leakage was several seconds, suggesting that the direct effects of irradiation had been negligible. Thus, C-LAI is both a safer and simpler method of root canal cleaning that can treat multiple canals simultaneously with less damage of the laser irradiation on the periapical area, while ensuring the effectiveness of the irrigation.

In our present study, the observation of cavitation bubbles after a single pulse of irradiation revealed differences in the duration and distance between the bubbles generated at the fiber tip and the subsequent bubbles that appeared in the center of the root canal, indicating a movement speed of about 100 m/s. The velocity of the irrigation solution within the root canal following laser irradiation has been reported to within 3 m/s^28^ and the bubble displacement observed in this study is unlikely to have occurred due to the irrigation movement itself. Since it has been shown that the propagation velocity of pressure waves easily decreases to several hundred m/s in the gas–liquid mixed phase^31^, the bubble movement velocity observed in our present experiment is a valid condition for pressure waves and cavitation generation. This phenomenon of cavitation bubbles and physical reaction occurrences away from the fiber tip accords with the previously reported behavior of laser-induced cavitation in the liquid phase^32^. Hence, the appearance of subsequent bubbles in this study is considered to be cavitation that occurs due to the decrease in the pressure inside the root canal rather than the physical movement of the bubbles. This impact pressure is theoretically estimated to be in the order of several mPa^33,34^. Thus the biofilm can be easily displaced from the root canal wall upon impact. Although the impact pressure is predicted to be much smaller in the actual root canal, this phenomenon is considered to be the main reason for the biofilm removal effect of C-LAI observed in our current study.

The pulse duration of the C-LAI used in our present study was about 300 μsec, which is larger than the 50 μsec pulse duration used in PIPS. The Er:YAG laser is capable of tooth structure ablation with irradiation energy increases, thus PIPS is designed to have a narrow pulse duration to optimize the agitation of the irrigant without tooth ablation^15^. However, it was found previously that PIPS cannot easily generate a shock wave inside the root canal system^35^. Hence, with the long pulse duration used in our present study, we assumed that the increased irradiation energy contributed to the generation of a sufficient pressure wave for cavitation bubbles to occur. Thus, cavitation by laser irradiation can generate a physical force on the root canal wall and is expected to become a novel treatment technique for non-contact biofilm removal in the retreatment of refractory periapical periodontitis cases. In addition, C-LAI can be clinically applied as an effective, safe, and relatively simple root canal treatment technique because it can shorten the treatment time by cleaning multiple root canals simultaneously by positioning the fiber tip in the pulp chamber.

A notable limitation of our current study was that the pig tooth used has a root length similar to that found in human teeth, but has a wider root foramen of approximately 0.7–1.0 mm. As the volume in the root canal decreases, the velocity of the pressure wave propagation may also change, requiring the further investigation of optimization conditions such as output settings and the shape of the fiber tip. In addition, the exudation of the chemical solution from outside the apical foramen is a potential concern for LAI and PIPS. It will thus be necessary to further investigate the effects of C-LAI and I-LAI, both of which were found to be useful for biofilm removal in our present experiments, on possible effluent leakage beyond the apex.

Establishing more effective root canal cleaning methods will improve the success rates of root canal retreatments. Although additional investigations are needed to further optimize laser irradiation methodologies, and verify the safety of these approaches, our current evidence indicates that C-LAI can safely improve irrigant activation for better biofilm removal without the need for other complicated techniques.

## Conclusions

A non-contact debridement modality is required to establish a reliable biofilm removal technique for orthograde root canal retreatments. Our current report is the first to indicate that C-LAI can generate secondary cavitation in the root canal, which can lead to the removal of biofilm through the simple placement of an optic fiber inside the pulp chamber. The C-LAI method is thus likely to contribute substantially to the future development of endodontic treatments for patients who suffer from refractory apical periodontitis caused by an intraradicular biofilm.

## Supplementary Information

Supplementary Legends.

Supplementary Video 1.

Supplementary Video 2.

## Data Availability

All the datasets used and analyzed during the current study are available from the corresponding author on reasonable request.

## Abbreviations

LAI: Laser-activated irrigation
PIPS: Photon-induced photoacoustic streaming
NaOCl: Sodium hypochlorite
CNI: Conventional needle irrigation
PPS: Pulse per second
FPS: Frames per second
