# Supplementary material for: Er:YAG laser-induced cavitation can activate irrigation for the removal of intraradicular biofilm
Source: Sci Rep. 2022 Mar 22;12:4897. doi: 10.1038/s41598-022-08963-x (PMC8940933; doi:10.1038/s41598-022-08963-x)
Supplement: Supplementary file 1 — Supplementary Legends. [file 41598_2022_8963_MOESM1_ESM.docx]

Supplementary file 1

High speed capture of bubble generation and collapse after a single pulse of irradiation inside a single root canal model.

Supplementary file 2

High speed capture of bubble behavior over time in a two root canal model.
